# Abnormal Sleep, Circadian Rhythm Disruption, and Delirium in the ICU: Are They Related?

**DOI:** 10.3389/fneur.2020.549908

**Published:** 2020-09-18

**Authors:** Marietou Daou, Irene Telias, Magdy Younes, Laurent Brochard, M. Elizabeth Wilcox

**Affiliations:** ^1^Interdepartment Division of Critical Care Medicine, University of Toronto, Toronto, ON, Canada; ^2^Department of Medicine (Respirology), University Health Network, Toronto, ON, Canada; ^3^Department of Medicine (Critical Care Medicine), St. Michael's Hospital, Toronto, ON, Canada; ^4^Keenan Research Centre, Li Ka Shing Knowledge Institute, Toronto, ON, Canada; ^5^Sleep Disorders Centre, Winnipeg, MB, Canada

**Keywords:** ICU acquired delirium, sleep, circadian rhythm, intervention, mechanisms

## Abstract

Delirium is a syndrome characterized by acute brain failure resulting in neurocognitive disturbances affecting attention, awareness, and cognition. It is highly prevalent among critically ill patients and is associated with increased morbidity and mortality. A core domain of delirium is represented by behavioral disturbances in sleep-wake cycle probably related to circadian rhythm disruption. The relationship between sleep, circadian rhythm and intensive care unit (ICU)-acquired delirium is complex and likely bidirectional. In this review, we explore the proposed pathophysiological mechanisms of sleep disruption and circadian dysrhythmia as possible contributing factors in transitioning to delirium in the ICU and highlight some of the most relevant caveats for understanding the relationship between these complex phenomena. Specifically, we will (1) review the physiological consequences of poor sleep quality and efficiency; (2) explore how the neural substrate underlying the circadian clock functions may be disrupted in delirium; (3) discuss the role of sedative drugs as contributors to delirium and chrono-disruption; and, (4) describe the association between abnormal sleep-pathological wakefulness, circadian dysrhythmia, delirium and critical illness. Opportunities to improve sleep and readjust circadian rhythmicity to realign the circadian clock may exist as therapeutic targets in both the prevention and treatment of delirium in the ICU. Further research is required to better define these conditions and understand the underlying physiologic relationship to develop effective prevention and therapeutic strategies.

## Introduction

Delirium is a syndrome characterized by acute brain failure typically arising over hours or few days that leads to a change in mental state (1–3). It primarily results in disturbances in attention (the ability of directing, focusing, sustaining, and shifting attention) and awareness of the environment (3). Prevalence can be as high as 80% in elderly patients receiving mechanical ventilation (4, 5). Delirium is associated with significant morbidity and mortality in the intensive care unit (ICU). Risk factors for delirium include acute illness, coma, more severe illness, emergency surgery, polytrauma, and mechanical ventilation; patient characteristics that are related to a higher risk of developing delirium include age and chronic conditions (e.g., dementia, hypertension) (6, 7). Environmental characteristics specific to the ICU and related to sleep and circadian rhythm disruption (8) may worsen symptoms. Such factors include the lack of normal variability in light-dark cycle (9), noise (10), the use of mechanical ventilation (11, 12), and need for continuous infusions of sedative drugs (13). The available literature suggests that there may be a close relationship between delirium, sleep, circadian rhythm, and critical illness, however, no causal pathway has been yet clearly described or the directionality of the relationship understood.

## Association Between Sleep Disturbances and Delirium

Sleep architecture changes throughout an individual's lifespan to support proper development and physiological function (14, 15). Normal sleep architecture varies among individuals but is made up of cycles of rapid eye movement (REM) and the 3 stages of non-REM (NREM) sleep; as an example, “normal” sleep in a healthy adult might be made up of 2–5% stage 1, 45–55% stage 2, 3–15% stage 3, or slow wave sleep and 20–25% REM (16). Transition from wake to sleep onset occurs within 10–20 min and the first period of REM typically occurs within 90–120 min. Poor sleep is associated with both neuropsychological and cognitive impairment (17). In a study by Zhou et al. (18) induced sleep deprivation in 13 young healthy men (mean age 23 yrs.) was associated with impairment in both attention and psychomotor vigilance. Further, in a study of 66 healthy adult volunteers chronic sleep deprivation was associated with increased reaction time to visual stimulus; experienced impairment was dose-dependent and variable time to recovery to baseline cognition was seen between individuals once normal sleep restored (19).

Poor sleep is common in the ICU (20, 21). Critically ill patients experience increases in stages 1 and 2 sleep with frequent arousals and awakenings. Further, they are less likely to transition into stage 3 or slow wave sleep or REM sleep (22–24). Poor sleep has been associated with delirium and other outcomes such as length of stay and long-term cognitive impairment (25–28). Additionally, there is a general believe among ICU clinicians that poor sleep is a risk factor for delirium as shown in a recent global survey, 97% of 1,223 ICU physicians and nurses (29). Moreover, the 2018 Clinical Practice Guidelines for Pain, Agitation, and Delirium (PAD), from the Society of Critical Care Medicine recommended using a sleep-promoting, multicomponent protocol in critically ill adults based on the pooled analysis of three observational before-after studies demonstrating an overall reduction in the prevalence of delirium when sleep promoting interventions were used (30, 31). Although a direct relationship between ICU delirium and sleep has yet to be shown, in long-term follow-up, total duration of ICU delirium was found by Altman et al. (32) to be significantly associated with increased sleep disturbance at long-term follow-up (mean 5 months after hospital discharge).

## Neural Networks Involved in Circadian Dysrhythmia and Delirium

Two primary processes, the homeostatic process (Process S) interacts with the Process C, controlled by the circadian pacemaker to control the normal sleep-wake cycle, with time courses regulated from physiological and behavioral variables (33). The suprachiasmatic nucleus (SCN) acts as a central pacemaker coordinating daily physiological and behavioral cycles. The SCN is a neural network that entrains peripheral cellular clocks across the body achieving circadian control of behavior, neuroendocrine and autonomic signals in target tissues (34).

The circadian rhythm appears to be disrupted commonly in critically ill patients (35–40). It is hypothesized that chronodisruption may be associated with ICU-acquired delirium. Alterations in the structure and function of specific neural networks represent potential substrates for this relationship. Among patients with impaired consciousness during vegetative or comatose states resting state functional magnetic resonance imaging (MRI) showed a reduction in connectivity of posteromedial and anteromedial cortices as well as temporoparietal junctions with worsening impairment in consciousness (41). The posteromedial cortex, including the posterior cingulate cortex is responsible for maintenance of wakefulness and consciousness (42). In 22 medical inpatients with delirium, Choi et al. (43) found abnormalities in functional connectivity of the posterior cingulate cortex, intralaminar thalamic nuclei, and mesencephalic networks, including the nucleus basalis and the ventral tegmental area, part of the ascending reticular activating system (ARAS), during episodes of delirium. Interestingly, abnormal resting state connectivity of the SCN, as assessed by functional MRI, has been identified by Kyeong et al. (44) as a possible substrate for chronodisruption in 34 delirious patients admitted to various inpatient units for various medical conditions. In this study, abnormal resting state connectivity between the SCN, cortical nodes including the posteromedial/anteromedial cortices, and temporoparietal junctions, and subcortical regions was seen in delirious patients (44).

An important hallmark of delirium is decreased activity of the ARAS (5): a neuronal network located in the brainstem that controls wake, sleep and dreaming states through connections with the SCN and releasing acetylcholine during the individual's active phase (45). In delirium, due to transient hypoxic states, the depletion of acetylcholinergic projections and the dopaminergic overproduction from the ARAS result in alertness and attention disturbances (5, 46). Normal circadian distribution of sleep-wakefulness states has been attributed to the intimate relationship between the SCN and cholinergic neurons within the ARAS such as the locus coeruleus (47, 48). However, despite consistently described alterations in melatonin metabolism in critically ill patients (36, 40, 49, 50), abnormalities in circadian connectivity seen on neuroimaging studies were not associated with measured melatonin levels.

## Biochemical Substrate of Sleep Disturbances, Circadian Dysrhythmia, and Delirium in Critical Illness

**Figure 1:**
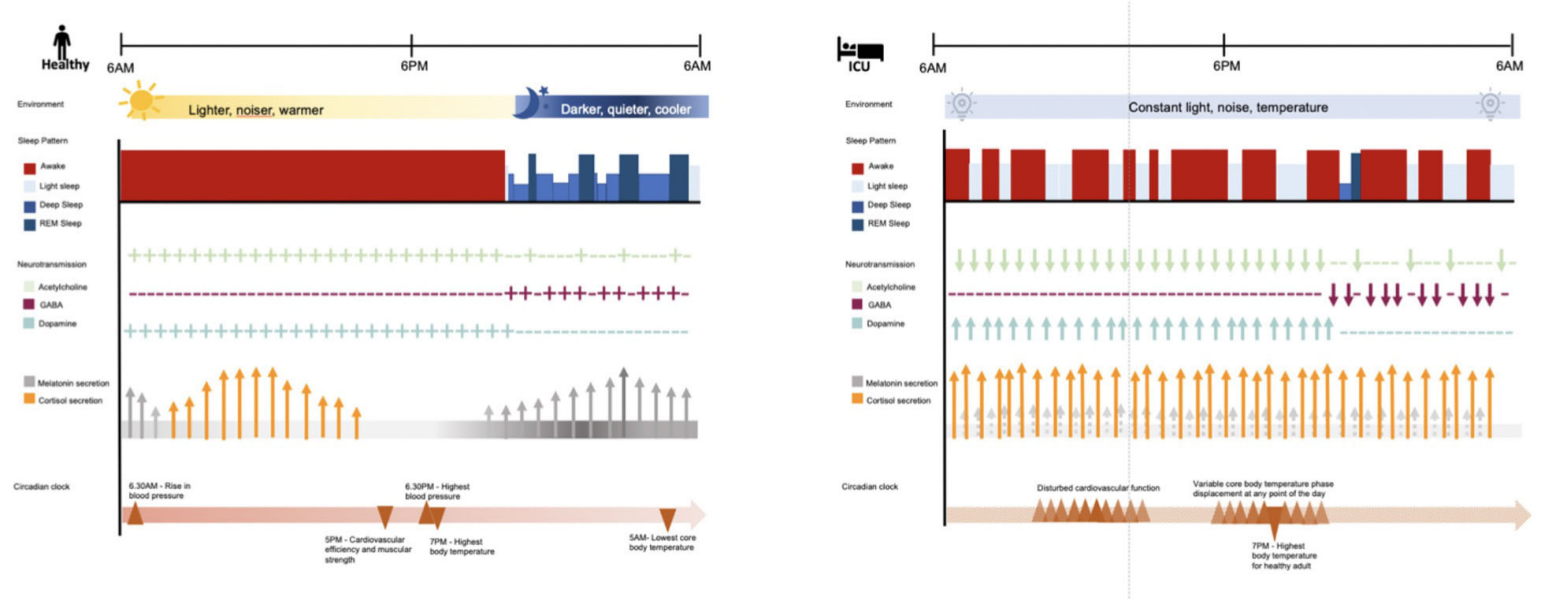
Comparison of sleep and circadian rhythms in healthy adult and adult with critical illness in the ICU. In a healthy adult, the circadian clock is synced to the daily external cycle of changing light, sound, and temperature. The sleep stage of a healthy adult occurs during the night and is composed of 2–5% light sleep, 48–70% deep sleep, 20–25% rapid eye movement (REM) sleep. REM sleep reoccurs in cycles of 90–120 min. Acetylcholine is predominantly discharged during wakefulness and REM sleep; while GABAergic activity is predominant during deep sleep. Dopaminergic activity promotes alertness and reduces sleep. Melatonin secretion starts around 9.30 pm (dim light melatonin onset) and stops around 7.30 am, peaking around 3 am. Cortisol secretion starts in the early morning, peaks around 10 am in correspondence to the time of highest alertness, and keeps declining gradually throughout the day, and the night. Overall, these processes coordinate physiological functions including cardiovascular functions and temperature. In contrast, the intensive care unit (ICU) constant artificial environment disrupts the daily cycle of circadian functions. In the ICU critically ill patients, compared to healthy adults, present equal to normal sleep time in the course of 24 h but the majority of it consists of light sleep; 50% of sleep time is distributed during the day and disturbed by frequent arousals. For instance, benzodiazepine decrease sleep latency, slow wave sleep (SWS) and REM sleep duration and frequency; propofol suppresses SWS EEG bursts; opioids alter REM sleep; while, dexmedetomidine improve stage 2 and sleep efficiency by shifting 75% of sleep to nighttime. In ICU patients, the acute stress environment has been associated with decreased GABAergic and cholinergic transmission, and increased dopaminergic transmission, impaired melatonin secretion and increased cortisol production, along with displacement of physiological functions normally coordinated by the circadian clock. These disturbances have been associated with symptoms of delirium. ICU, intensive care unit; REM, rapid eye movement; GABA, gamma-aminobutyric acid.

Abnormal sleep architecture (20), circadian dysrhythmia (8), and delirium (51–53) are all common in the ICU. Disturbance in sleep-wake cycle, alertness and altered sensorium often have multiple possible etiologies and likely share attributable pathophysiological mechanisms (See Figure 1).

### Gamma-Aminobutyric Acid(GABA)-Ergic Mechanisms

The upregulation of GABA-A receptors, either through an increase in synthesis of endogenous GABA agonists or stimulation from exogenous GABA agonists, has been implicated in delirium (54, 55). GABA-ergic agents are thought to destabilize neurons preventing sleep transition (55), and upregulate inhibitory tone in the central nervous system contributing to neural connectivity and cognitive disintegration (56). Both benzodiazepines and propofol, commonly administered sedatives in the ICU, mediate their effect by modulating the effects of GABA. Zaal et al. (13) demonstrated that continuous infusion as compared to intermittent bolus dosing was associated with delirium; suggesting that higher doses or greater exposure is an important factor in transitioning to delirium. Although the interpretation of sleep in the ICU can be difficult in sedated patients, benzodiazepines typically increase total sleep time through prolonging stage 2 NREM, while suppressing both stage 3 NREM or slow wave sleep and REM (57). Propofol has been shown to worsen overall sleep architecture; specifically, suppressing REM sleep (58).

In contrast to the potentially deleterious effects of GABA agonists, the use of the alpha-2 agonist dexmedetomidine has been shown to be relatively effective in decreasing the daily prevalence of delirium in mechanically ventilated ICU patients (59). Dexmedetomidine is a highly selective alpha-2 agonist that facilitates both sedation and analgesia, without much respiratory depression. It appears to be particularly effective in lowering the daily prevalence and duration of delirium when compared with benzodiazepines, such as lorazepam, the comparator in the randomized, double-blind MENDS trial (60). The daily prevalence of delirium in dexmedetomidine-treated patients was also significantly lower when compared to a midazolam group in the randomized, double-blind SEDCOM trial (61). Recently, the prophylactic use of nocturnal dexmedetomidine was shown to reduce the incidence of delirium in the SKY-DEX trial (62). In the DahLIA, placebo-controlled trial (63), 74 mechanically ventilated patients with agitation and delirium were randomized to dexmedetomidine or placebo; patients treated with dexmedetomidine had increased ventilator-free hours at 7 days (median, 145 vs. 128 h) and faster resolution of their delirium symptoms (median, 23 vs. 40 h). The effect of dexmedetomidine on delirium prevention may be mediated both through its reduction in glutamate release, as glutamate toxicity has been previously associated with the development of delirium (64), but also through its decreased GABA receptor modulation or cholinergic receptor activity when compared to other commonly used sedatives (65). Furthermore, dexmedetomidine through the stimulation of alpha-2 receptors and resultant inhibition of noradrenergic neurons in the locus coeruleus and disinhibition of GABA neurons in the ventrolateral preoptic nucleus it may promote more natural sleep in the ICU environment (66).

### Melatonergic Mechanisms

Disturbed melatonergic activity is also implicated in delirium pathogenesis. Risk factors for delirium including pre-existing cognitive impairment, old age, and psychotropic medication use are all associated with impaired melatonergic function. Melatonin deficiency and abnormal secretion contribute to impairment of the sleep-wake cycle. Perras et al. (67) report that the normal response of melatonin secretion to changes in light and darkness is impaired in critically ill patients suggesting a dysregulation of the mechanism of melatonin secretion or a shift in the circadian clock phase in the SCN. Altered urinary levels of the melatonin metabolite 6-sulfatoxymelatonin (6-SMT) has been reported in delirious patients as compared to patients who were not delirious; levels were elevated in hypoactive patients and lower in hyperactive patients (68). Urinary 6-SMT exhibited loss of circadian rhythmicity with no daytime decline in septic patients (36), a frequently present in critical illness. Recently, Li et al. (69) measured plasma levels of melatonin, TNF-α, IL-6 and messenger RNA of the circadian genes Cry-1 and Per-2 for 24-h in septic and non-septic ICU patients (*n* = 22). Altered circadian rhythm of melatonin secretion, reduced expression of Cry-1 and Per-2, and elevated levels of TNF-α and IL-6 were again seen in patients with sepsis (69).

### Hypothalamic-Pituitary-Adrenal-Stress Axis

Hypothalamic-pituitary-adrenal-stress axis (HPA) dysregulation might also be related to incident delirium and circadian dysrhythmia and is intimately related to the SCN and melatonergic functions. Elevated plasma cortisol levels are associated with increased delirium risk after both cardiac- (70) and non-cardiac surgery (71). Pearson et al. (72) found that in post-operative patients over the age of 60 years with acute hip fracture, cortisol CSF levels were elevated in those who developed delirium as compared to those who did not. It has been postulated that aberrant stress responses related to disrupted function in the limbic-HPA axis and its interaction with the inflammatory response may be responsible for the increased risk of delirium with age as well as pre-existing cognitive impairment (73). In a cohort of ICU patients with severe sepsis and septic shock (*n* = 140), plasma cortisol level between 6 and 12 h post hemodynamic stabilization (odds ratio [OR]: 2.3, 95% CI 2.0–3.2; *p* = 0.02) and the combination of older age and plasma cortisol level (OR: 1.0, 95% CI 1.0–1.9; *p* = 0.04) were associated with increase delirium risk (74).

## Strategies to Improve Sleep and Realign Circadian Rhythm in the ICU

Numerous opportunities exist to improve sleep and re-entrain circadian rhythm in the ICU. Easily modifiable risk factors associated with disruption of sleep and circadian rhythm such as mechanical ventilation strategies paired with sedation stewardship and multicomponent sleep improvement strategies in the ICU may help to reduce delirium duration or severity and facilitate recovery from critical illness (75) (See Table 1).

**Table 1:** Strategies to improve sleep and realign circadian rhythm in the ICU.

**Intervention**	**Mechanism**	**Evidence**
Increase light-dark contrast in the ICU	Loss of exposure to natural light is associated with circadian rhythm disruption that may impact incident delirium and outcomes in critically ill patients	Existing evidence does not support routine light therapy in isolation, the optimal dose, timing, and patient population require further investigation and it may be that light therapy is best deployed as part of a multicomponent strategy
Melatonin therapy	Potential role of melatonin to regulate the circadian distribution of sleep and prevent ICU-acquired delirium	General interest in the potential prophylactic use of melatonin but no current recommendations exist supporting the exogenous administration of melatonin to treat ICU-acquired delirium • Recent meta-analysis indicated that the peri-operative supplementation of melatonin agonists reduced the risk of transitioning into delirium to 37% compared to placebo or no treatment (*p =* 0.006) (76) • Investigators are currently evaluating the use of melatonin as a prophylactic agent for delirium prevention in the ICU (77)
Mechanical ventilation	Relationship between mechanical ventilation, sleep disturbances, and circadian dysrhythmia not well-understood and likely complex as relate to the use of sedative agents	Attention should be paid to reducing over-assistance with pressure support ventilation and, as available, clinicians should consider modes such as proportional assist-ventilation, and neurally adjust ventilatory assist

### Mechanical Ventilation

Relationship between mechanical ventilation, sleep disturbances and circadian dysrhythmia are not well-understood as they are complex and likely associated with variation in the administration of sedative agents and subsequent patient-ventilator asynchrony management (8). However, this relationship strongly influences the weaning process as sleep disturbances and delirium are associated with greater duration of mechanical ventilation and prolonged ICU length of stay (78–80). An opportunity must therefore exist for intervention with the aim of modifying ventilator settings and practices for improving sleep and circadian rhythmicity.

Sedation is commonly used to help patients tolerate mechanical ventilation. The 2018 PADIS Clinical Guidelines recommend the use of light levels of sedation (Richmond Agitation Sedation Score of −2 to +1) during invasive mechanical ventilation (30). This inevitably results in patients breathing spontaneously during assisted ventilation and resultant abnormal patient-ventilator interactions named asynchronies. The typical reaction of physicians to the presence of asynchronies involves the administration of additional sedation boluses or increasing the dose of sedative infusions which can significantly affect sleep architecture, circadian rhythmicity and delirium risk. However, not all asynchronies are “improved” (81) with sedation (82, 83). Therefore, an understanding of the mechanisms leading to the experienced asynchrony and personalized adjustment of ventilator settings (81) might spare a patient from further exposure to sedative agents, possibly mitigating further risk of sleep and circadian rhythm disruption. The titration of pressure support settings during non-invasive ventilation by adjusting the amount of support to meet the needs of the patient has previously been shown to decrease the number of asynchronies and improve sleep architecture in patients with chronic neuromuscular diseases (84).

Caution however needs to be exercised as over-assistance during pressure support can alternately lead to central apneas with resultant poor sleep, characterized by frequently awakenings and arousals leading to greater sleep fragmentation (85, 86). Ventilatory support in excess to a patient's metabolic need results in hyperventilation where CO_2_ levels decrease below the apnea threshold (87). To eliminate over-assistance, which can also lead to other adverse outcomes such as disuse diaphragmatic atrophy (88), the amount of ventilatory support needs to be decreased (89). Alternatively, assist-controlled modes with a back-up respiratory rate can also be used to prevent apnea events which has been shown to reduce sleep fragmentation (86). Using assist-controlled modes however does not eliminate the risk of disuse diaphragmatic atrophy and might create the false impression that patients are not ready for liberation, potentially delaying extubation. Proportional modes of ventilation [i.e., proportional assist-ventilation [PAV+] and neurally adjust ventilatory assist [NAVA]] can be used to deliver pressure proportional to the patient's instantaneous efforts in terms of amplitude and timing which avoids over- and under-assistance and improves synchrony. The use of these modes has systematically been shown to decrease asynchronies (82, 90, 91) and improve sleep quality in cross-over studies of small size (92, 93).

### Multicomponent Strategies

Recently a review by Flannery investigated whether interventions targeted at improving sleep in the ICU were associated with reductions in ICU delirium (94). Six of the ten identified studies demonstrated a statistically significant reduction in the incidence of ICU delirium associated with sleep intervention. Unfortunately, although sleep interventions seem to be a promising approach for improving delirium and related outcomes (e.g., ICU length of stay) conclusions are limited by confounding, variable methodology and bias issues. Further study is therefore needed. Based on available data, potentially effective interventions might include a combination of dexmedetomidine (62), oral melatonin (95), and cognitive behavioral sleep therapy (96) using modified polysomnography to quantify sleep quality and quantity (25, 78) and understand to which extent sleep actually affects delirium occurrence.

## Conclusions

Despite advances in our understanding of the sleep-wake cycle its association with the underlying mechanism of delirium, and how both influence ICU patient outcome, significant knowledge gaps exist in understanding the relationships between sleep, circadian rhythm and delirium. Hypotheses for mechanistic relationships between the sleep-wake cycle and delirium have largely been derived from studies of non-ICU patients. A better understanding of mechanisms would guide the development of new methods for prevention and treatment that consequently may improve short- and long-term outcomes of ICU survivors.

## Author Contributions

MW, MD, and IT wrote the first draft and revised the manuscript. All authors revised and approved the final manuscript.

## Conflict of Interest

The authors declare that the research was conducted in the absence of any commercial or financial relationships that could be construed as a potential conflict of interest. The reviewer GB declared a shared affiliation, with no collaboration, with three of the authors IT, LB, and MW to the handling Editor.
